# Screening Programs for SARS-CoV-2 Infections on a University Campus — Austin, Texas, September 30–November 30, 2020 

**DOI:** 10.15585/mmwr.mm7035a4

**Published:** 2021-09-03

**Authors:** Kayleigh J. Nerhood, Emily R. James, Allen Hardin, James E. Bray, Terrance S. Hines, Amy E. Young, Darlene Bhavnani

**Affiliations:** ^1^Dell Medical School, University of Texas at Austin; ^2^University of Texas at Austin Athletics; ^3^University Health Services, University of Texas at Austin.

Colleges and universities in the United States have relied on various measures during the COVID-19 pandemic to prevent transmission of SARS-CoV-2, the virus that causes COVID-19, including implementing testing programs (*1*–*3*). These programs have permitted a safer return to campus for students by identifying infected persons and temporarily isolating them from the campus population (*2*,*3*). The University of Texas at Austin (UT Austin) implemented COVID-19 prevention measures in Fall 2020* including the following testing programs: clinic-based diagnostic testing, voluntary community screening, and targeted screening (testing of specific student populations in situations of increased transmission risk). During September 30–November 30, 2020, UT Austin students participated in tests for SARS-CoV-2, which resulted in the detection of 401 unique student cases of COVID-19 from among 32,401 tests conducted.^†^ Among students who participated in one targeted screening program for students attending campus events, 18 (37.5%) of 48 infected students were asymptomatic at the time of their positive test result compared with 45 (23%) of 195 students identified through community testing and nine (5.8%) of 158 students identified through clinic-based testing. Targeted screening also identified a different population of students than did clinic-based and community testing programs. Infected students tested through targeted screening were more likely to be non-Hispanic White persons (chi square = 20.42; p<0.03), less likely to engage in public health measures, and more likely to have had interactions in settings where the risk for SARS-CoV-2 transmission is higher, such as restaurants, gyms, and residence halls. In addition to clinic-based SARS-CoV-2 testing at colleges and universities, complementary testing programs such as community and targeted screening might enhance efforts to identify and control SARS-CoV-2 transmission, especially among asymptomatic persons and disproportionately affected populations that might not otherwise be reached.

During September 30–November 30, 2020, UT Austin employed the following SARS-CoV-2 testing programs: 1) clinic-based diagnostic testing administered by University Health Services for persons who were symptomatic or reported exposure to SARS-CoV-2 (clinic-based testing); 2) Proactive Community Testing, which involved voluntary screening of asymptomatic persons offered at several fixed or rotating sites on-and-off campus (community testing); and 3) targeted screening of specific student populations in situations of increased transmission risk. One targeted screening program focused on Big Ticket holders, students with season tickets to athletic events. These events are large gatherings that might involve several SARS-CoV-2 infection risk factors such as several hours of possible exposure, the potential for crowding, and behaviors such as singing and shouting.^§^ Students were tested up to 3 days before each event. Either a negative test result or proof of previous SARS-CoV-2 infection 14–90 days before the event was required for entry. Community testing and targeted screening programs were provided to students at no cost; clinic-based tests were billed to students’ insurance. Cases were identified through clinic-based testing using SARS-CoV-2 nucleic acid amplification tests (NAATs), including reverse transcription–polymerase chain reaction (RT-PCR) or isothermal NAAT (ID NOW [Abbott] or Aptima SARS-CoV-2 Assay [Hologic]). Community testing used a Clinical Laboratory Improvement Amendments (CLIA)-certified RT-PCR test performed at a UT laboratory, and testing for Big Ticket holders used an antigen test (Sofia SARS Antigen Fluorescent Immunoassay [Quidel Corporation])^¶^ or UT’s CLIA-certified RT-PCR test. Test results were reported to Dell Medical School at UT Austin, which was delegated by Austin Public Health to conduct contact tracing. Contact tracers interviewed infected persons to identify close contacts** during their infectious period,^††^ and collected exposure details, including dates, proximity, location, duration of exposure, and mask use. Characteristics, symptom status, isolation practices, and case investigation outcomes among students with COVID-19 were assessed; statistical comparisons among cases identified by the different testing programs were performed using chi square tests or one-way ANOVA in Python (version 3.7.9; Python Software Foundation) using the SciPy statistical package (version 1.5.4; Python Software Foundation); p values <0.05 were considered statistically significant. This study was reviewed by a UT Institutional Review Board and deemed to not be human subjects research. This activity was reviewed by CDC and conducted consistent with applicable federal law and CDC policy.^§§^

Among 32,401 tests of UT Austin students, 401 unique COVID-19 cases were identified (Table 1); 3,044 tests were done through clinic-based testing, 25,042 through community testing, and 4,314 through testing of Big Ticket holders. Among one targeted screening program for Big Ticket holders, 75% of infected students self-identified as non-Hispanic White persons, compared with 48.7% of infected students detected by community testing and 58.9% of infected students detected by clinic-based testing (chi square = 20.42; p<0.03). The proportion of non-Hispanic White students identified by each of the three testing programs was higher than that reported for the overall UT Austin student population^¶¶^ (38.9%; chi square = 177; p<0.001). UT contact tracers interviewed 85.5% of all infected persons. Among Big Ticket holders, 75% of infected persons were interviewed, 20.8% were unreachable by phone, and 4.2% stated they were unwilling to participate in the interview, a larger proportion of refusals than for community testing (1.0%) and clinic-based testing (0.6%).

**TABLE 1:** Demographic characteristics, symptom status, isolation practices, and case investigation outcomes among students with COVID-19, by testing program — University of Texas at Austin, September 30–November 30, 2020

Characteristic (no. with available information)	No. (%)			
	Total	Testing program		
		Big Ticket holder*	Community	Clinic-based
**Students in testing programs**	**401 (100)**	**48 (12.0)**	**195 (48.6)**	**158 (39.4)**
**Age, yrs, median (range)**	20 (18–29)	19.5 (18–22)	20 (18–28)	21 (18–29)
**Sex **(401)				
Male	187 (46.6)	19 (39.6)	86 (44.1)	82 (51.9)
Female	213 (53.1)	29 (60.4)	108 (55.4)	76 (48.1)
Unknown	1 (0.2)	0 (—)	1 (0.5)	0 (—)
**Race/Ethnicity **(401)				
White, non-Hispanic	224 (55.9)	36 (75.0)	95 (48.7)	93 (58.9)
Black, non-Hispanic	14 (3.5)	0 (—)	7 (3.6)	7 (4.4)
Asian, non-Hispanic	37 (9.2)	2 (4.2)	21 (10.8)	14 (8.9)
White, Hispanic	89 (22.2)	5 (10.4)	56 (28.7)	28 (17.7)
Multiracial	8 (2.0)	0 (—)	3 (1.5)	5 (3.2)
Unknown	29 (7.2)	5 (10.4)	13 (6.7)	11 (6.9)
**Outcomes of COVID-19 case investigations **(401)				
Interviewed	343 (85.5)	36 (75.0)	171 (87.7)	136 (86.1)
Unable to interview	53 (13.2)	10 (20.8)	22 (11.3)	21 (13.3)
Unwilling to participate	5 (1.2)	2 (4.2)	2 (1.0)	1 (0.6)
**Symptom status**				
Symptomatic	284 (70.8)	22 (45.8)	129 (66.2)	133 (84.2)
Asymptomatic	72 (18.0)	18 (37.5)	45 (23.1)	9 (5.7)
Unknown	45 (11.2)	8 (16.7)	21 (10.7)	16 (10.1)
**Patient isolation** (343)^†^				
Yes	317 (92.4)	29 (80.6)	156 (91.2)	132 (97.1)
No	23 (6.7)	5 (13.9)	14 (8.2)	4 (2.9)
Unknown	3 (0.9)	2 (5.6)	1 (0.6)	0 (—)
**Specimen collection relative to symptom** **onset**^§^ (274)				
Before symptom onset	28 (10.2)	3 (15.0)	18 (14.2)	7 (5.5)
On or after symptom onset	246 (89.8)	17 (85.0)	109 (85.8)	120 (94.5)
**Start of isolation relative to symptom onset**^§^ (274)				
Before symptom onset	42 (15.3)	0 (—)	15 (11.8)	27 (21.3)
On or after symptom onset	203 (74.1)	13 (65.0)	98 (77.2)	92 (72.4)
Unknown	29 (10.6)	7 (35.0)	14 (11.0)	8 (6.3)

* Screening targeted to students who held season tickets to athletic events.

^†^ Population limited to persons who were interviewed.

^§^ Population limited to persons who were interviewed and symptomatic.

Approximately 38% of cases among Big Ticket holders occurred in persons who were asymptomatic at the time of their positive test results, compared with 23% identified through community testing and 6% through clinic-based testing (chi square = 35; p<0.001). Higher proportions of infected students from the Big Ticket and community testing programs were tested before symptom onset (15.0% and 14.2%, respectively) compared with clinic-based testing (5.5%); however, these differences were not statistically significant. Infected persons detected through testing of Big Ticket holders were less likely to have isolated after receiving a positive result (80%) than were those identified through community (91.2%) and clinic-based testing (97.1%).

Among 195 cases detected through community testing and 48 through testing of Big Ticket holders, 120 (61.5%) and 35 (72.9%) persons, respectively had no previous engagement with community testing (Table 2). Among 40 asymptomatic infected persons who had no previous community testing history, the testing program for Big Ticket holders identified a higher proportion of asymptomatic cases than did community testing (31.4% versus 24.2%; chi square = 7.53; p = 0.02).

**TABLE 2:** Symptom status* of student COVID-19 cases detected by community testing and testing for Big Ticket holders,^†^ stratified by previous history with community testing — University of Texas at Austin, September 30–November 30, 2020

Symptom status	Total N = 243	No. (%)			
		History of community testing			
		No n = 155		Yes^§ ^n = 88	
		Community n = 120	Big Ticket holder^¶^ n = 35	Community n = 75	Big Ticket holder n = 13
Asymptomatic	63 (25.9)	29 (24.2)	11 (31.4)	16 (21.3)	7 (53.8)
Symptomatic	151 (62.1)	76 (63.3)	17 (48.6)	53 (70.7)	5 (38.5)
Unknown	29 (11.9)	15 (12.5)	7 (20.0)	6 (8.0)	1 (7.7)

* Symptom status reported at time of case investigation.

**^†^** Excluding cases detected by the University Health Services clinic-based testing.

^§^ Infected persons had at least one COVID-19 test via community testing at any time before their positive result and during the study period.

^¶^ Students who held season tickets to athletic events.

A similar average number of close contacts was reported by infected persons identified from testing of Big Ticket holders (2.6 per person), community testing (3.1), and clinic-based testing (2.7) (p = 0.5). The most frequently reported exposure location among all testing programs was household (44%), defined as a shared living space (including a shared room or suite in a residence hall) (Table 3). The second most common exposure location identified through community and clinic-based testing was private residence or apartment visits (24% and 29%, respectively). In contrast, restaurants (22%) and residence halls (16%) were the next most common exposure locations among infected persons identified through testing for Big Ticket holders. These persons also reported a higher proportion of exposures in fitness or recreational facilities (6%) than did persons identified through community testing (3%) and clinic-based testing (1%), and a lower proportion of exposures outdoors (2% versus 13% and 6%, respectively; chi square = 145; p<0.001). Across all programs, most exposures were characterized by one or both students not wearing a mask (91.4% of Big Ticket holders and 87.9% of those who received community and clinic-based testing) (chi square = 1.1; p = 0.3). Contact tracers provided counseling to both infected persons and close contacts on appropriate mask use to prevent future exposures or reinfection.

**TABLE 3:** Location of exposure* among persons with COVID-19^†^ and their contacts, by testing program — University of Texas at Austin, September 30–November 30, 2020

Location	Testing program, no. (%)			
	Total	Big Ticket holder^§^	Community	Clinic-based
	N = 1,147	n = 123	n = 603	n = 421
Household	502 (44)	42 (34)	250 (41)	210 (50)
Restaurant	74 (6)	27 (22)	34 (6)	13 (3)
Residence hall visit	53 (5)	20 (16)	25 (4)	8 (2)
Private residence visit	292 (25)	17 (14)	145 (24)	130 (31)
Fitness or recreational facility	32 (3)	7 (6)	20 (3)	5 (1)
Outdoor	105 (9)	2 (2)	77 (13)	26 (6)
Other	89 (8)	8 (7)	52 (9)	29 (7)

* If an infected person and a close contact interacted in multiple locations, contact tracers chose the most likely transmission site based on duration, proximity, ventilation, and mask use.

^†^ Population limited to persons who were interviewed and named close contacts.

^§^ Students who held season tickets to athletic events.

## Discussion

Clinic-based diagnostic testing is a valuable tool to detect SARS-CoV-2 infection, particularly among symptomatic persons; however, complementary testing programs might enhance case detection (*4*). At UT Austin, one targeted screening program (conducted before vaccine availability) that tested Big Ticket holders identified a significantly higher proportion of asymptomatic persons than did clinic-based diagnostic testing at University Health Services (as expected), and voluntary screening through Proactive Community Testing. This targeted testing program resulted in the identification of potential asymptomatic spreaders, who might not have been detected through clinic-based or community testing (*5*).

Targeted screening of Big Ticket holders identified a different population from those identified by community and clinic-based testing: students who were predominantly non-Hispanic White and less likely to participate in voluntary public health prevention strategies including community testing, early isolation, and contact tracing. These Big Ticket holders also had more exposures in restaurants, a documented risk factor for SARS-CoV-2 infection (*6*), and in fitness or recreational facilities, locations of several large outbreaks (*7*). They also interacted more within residence halls, which include shared facilities and social areas; risks for transmission in these settings might be similar to those experienced in long-term care facilities (*1*,*8*,*9*).

The findings of this study are subject to at least six limitations. First, this study analyzed only one targeted testing program among students aged 18–29 years. Assessment of other targeted programs to include a broader age range might alter these findings. Second, both antigen tests and NAATs were used in testing of Big Ticket holders with different turnaround times for results (<2 hours for antigen tests and 24–48 hours for NAATs), which might have affected infected persons’ isolation timing and number of close contacts during their infectious period. Differences in NAAT and antigen test sensitivity might have also affected case ascertainment, with antigen tests potentially missing contagious persons and NAAT potentially detecting persons no longer infectious (*10*). Antigen tests were not confirmed with NAATs, because rapid results were required to exclude potentially infectious persons from next-day events. Third, symptom status was self-reported and recorded at the time of the interview; therefore, the number of asymptomatic cases could have been overestimated. However, targeted screening would have still succeeded in identifying presymptomatic cases. Fourth, symptoms caused by allergies, stress, or other infectious diseases might have been incorrectly attributed to COVID-19, inflating the number of symptomatic cases, particularly among those from clinic-based testing. Fifth, whether symptoms that started the day of the test began before or after the test is not known, which might underestimate the proportion of students who were tested before symptom onset. Finally, the higher proportion of infected Big Ticket holders who were unavailable or unwilling to participate in contact tracing compared with the other testing program groups, might have affected comparisons of symptom status, isolation, and exposures to close contacts.

Screening tests are an important part of risk-reduction strategies on college and university campuses and in other congregate settings. Targeted testing in this university effort facilitated reaching and identifying infected persons who might not have been detected through other testing measures. Therefore, targeted testing might be used as a complement to diagnostic and voluntary community screening measures on college and university campuses, particularly in high-risk or large gatherings such as university athletic events or graduation ceremonies. However, if antigen tests are used for asymptomatic screening, confirmatory NAATs of positive results should be considered if the likelihood of SARS-CoV-2 infection is low, such as if the person has no known exposure (*10*). Further research on targeted testing in other potential high-risk settings such as residence halls is warranted, especially if a large proportion of these persons are unvaccinated, or as variants of SARS-CoV-2 emerge.

**Summary**

**What is already known about this topic?**

University testing programs have permitted a safer return of students to campus by identifying persons with COVID-19 and temporarily isolating them from the campus population.

**What is added by this report?**

Targeted screening identified 48 cases of COVID-19 during September–November 2020, 18 (38%) of which were in asymptomatic persons. This population of infected students was demographically different from those identified through other testing programs, more risk-tolerant, and less willing to participate in public health prevention activities.

**What are the implications for public health practice?**

In addition to clinic-based diagnostic SARS-CoV-2 testing at colleges and universities, a complementary strategy of community and targeted screening programs might enhance efforts to identify and control transmission of COVID-19.

## Footnotes

*
https://protect.utexas.edu/

† A COVID-19 case was defined as a positive SARS-CoV-2 nucleic acid amplification test or antigen test result.

§
https://www.cdc.gov/coronavirus/2019-ncov/community/large-events/considerations-for-events-gatherings.html

¶
https://www.fda.gov/media/137884/download

** Close contact was defined as being within 6 ft of a person with laboratory-confirmed or probable COVID-19 infection for a cumulative total of ≥15 minutes during a 24-hour period; or having physical contact with; or sharing living spaces such as bedrooms, bathrooms, or kitchens. https://www.cdc.gov/coronavirus/2019-ncov/php/contact-tracing/contact-tracing-plan/appendix.html

†† The infectious period was estimated to begin 2 days before symptom onset and end ≥10 days after symptom onset or positive test result, as long as other symptoms (except loss of taste or smell) were improving and the patient had been fever-free for 24 hours without fever-reducing medication, according to CDC guidance. https://www.cdc.gov/coronavirus/2019-ncov/php/contact-tracing/contact-tracing-plan/investigating-covid-19-case.html

§§ 45 C.F.R. part 46, 21 C.F.R. part 56; 42 U.S.C. Sect. 241(d); 5 U.S.C. Sect. 552a; 44 U.S.C. Sect. 3501 et seq.

¶¶
https://www.utexas.edu/about/facts-and-figures

## References

[1] Wilson E, Donovan CV, Campbell M, Multiple COVID-19 clusters on a university campus—North Carolina, August 2020. MMWR Morb Mortal Wkly Rep 2020;69:1416–8. 10.15585/mmwr.mm6939e3 33001871 PMC7537562

[2] Fox MD, Bailey DC, Seamon MD, Miranda ML. Response to a COVID-19 outbreak on a university campus—Indiana, August 2020. MMWR Morb Mortal Wkly Rep 2021;70:118–22. 10.15585/mmwr.mm7004a3 33507894 PMC7842813

[3] Denny TN, Andrews L, Bonsignori M, Implementation of a pooled surveillance testing program for asymptomatic SARS-CoV-2 infections on a college campus—Duke University, Durham, North Carolina, August 2–October 11, 2020. MMWR Morb Mortal Wkly Rep 2020;69:1743–7. 10.15585/mmwr.mm6946e1 33211678 PMC7676642

[4] Paltiel AD, Zheng A, Walensky RP. Assessment of SARS-CoV-2 screening strategies to permit the safe reopening of college campuses in the United States. JAMA Netw Open 2020;3:e2016818. 10.1001/jamanetworkopen.2020.16818 32735339 PMC7395236

[5] Buitrago-Garcia D, Egli-Gany D, Counotte MJ, Occurrence and transmission potential of asymptomatic and presymptomatic SARS-CoV-2 infections: a living systematic review and meta-analysis. PLoS Med 2020;17:e1003346. 10.1371/journal.pmed.1003346 32960881 PMC7508369

[6] Fisher KA, Tenforde MW, Feldstein LR, ; IVY Network Investigators; CDC COVID-19 Response Team. Community and close contact exposures associated with COVID-19 among symptomatic adults ≥18 years in 11 outpatient health care facilities—United States, July 2020. MMWR Morb Mortal Wkly Rep 2020;69:1258–64. 10.15585/mmwr.mm6936a5 32915165 PMC7499837

[7] Lendacki FR, Teran RA, Gretsch S, Fricchione MJ, Kerins JL. COVID-19 outbreak among attendees of an exercise facility—Chicago, Illinois, August–September 2020. MMWR Morb Mortal Wkly Rep 2021;70:321–5. 10.15585/mmwr.mm7009e2 33661859 PMC7948936

[8] Lewis M, Sanchez R, Auerbach S, COVID-19 outbreak among college students after a spring break trip to Mexico—Austin, Texas, March 26–April 5, 2020. MMWR Morb Mortal Wkly Rep 2020;69:830–5. 10.15585/mmwr.mm6926e1 32614814 PMC7332093

[9] Kimball A, Hatfield KM, Arons M, ; Public Health – Seattle & King County; CDC COVID-19 Investigation Team. Asymptomatic and presymptomatic SARS-CoV-2 infections in residents of a long-term care skilled nursing facility—King County, Washington, March 2020. MMWR Morb Mortal Wkly Rep 2020;69:377–81. 10.15585/mmwr.mm6913e1 32240128 PMC7119514

[10] CDC. Interim guidance for antigen testing for SARS-CoV-2. Atlanta, GA: US Department of Health and Human Services, CDC; 2020. https://www.cdc.gov/coronavirus/2019-ncov/lab/resources/antigen-tests-guidelines.html

